# Beyond appearance: Can morphologically low‐grade euploid blastocysts yield successful pregnancies?

**DOI:** 10.1002/rmb2.12560

**Published:** 2024-01-18

**Authors:** Takahiro Suzuki, Chiharu Ishida, Yoko Yoshioka, Masae Kojima, Mikiko Tokoro, Noritaka Fukunaga, Yoshimasa Asada

**Affiliations:** ^1^ Asada Ladies Clinic Nagoya Aichi Japan; ^2^ Asada Institute for Reproductive Medicine Nagoya City Aichi Prefecture Japan

**Keywords:** blastocyst inner cell mass, fertilization in vitro, live birth, preimplantation diagnosis, single embryo transfer

## Abstract

**Purpose:**

The primary objective of this investigation is to evaluate how morphological quality affects the pregnancy outcomes in euploid embryos determined by preimplantation genetic testing for aneuploidies (PGT‐A). Concurrently, as a secondary objective, we aim to identify which specific aspects of morphological evaluation exert the most significant impact on these outcomes.

**Methods:**

A retrospective analysis of 451 single euploid embryo transfer cycles at our clinic was conducted. Embryos were evaluated based on the degree of blastocyst expansion, inner cell mass (ICM), trophectoderm (TE) morphology, and the day of blastocyst vitrification. Outcomes between morphologically low‐grade and high‐grade embryos were compared. Additionally, the study analyzed which morphological factors most influenced pregnancy outcomes.

**Results:**

Pregnancy outcomes were significantly lower in morphologically low‐grade blastocysts compared to high‐grade ones. Among the morphological evaluations, the ICM assessment was significantly associated with the live birth rate.

**Conclusion:**

Our study indicates that the morphological quality of euploid embryos, particularly the evaluation of the ICM, plays a crucial role in IVF‐ET success.

## Abstract 2

In this study, we investigated the impact of morphological evaluation on the success rates of in vitro fertilization and embryo transfer (IVF‐ET) in euploid embryos. Our results indicated significantly better pregnancy outcomes for embryos with morphologically high‐grade evaluations compared to those with low ones, with the assessment of the inner cell mass (ICM) being notably associated with live birth rates. This suggests the crucial role of morphological quality, especially ICM evaluation, in the success of IVF‐ET with euploid embryos.

## 1 INTRODUCTION

The success rate of in vitro fertilization and embryo transfer (IVF‐ET) procedures is fundamentally contingent upon the quality of the selected embryo. Therefore, precise evaluation and judicious selection of embryos—based on their likelihood to culminate in a successful pregnancy—emerge as critical factors. In this context, two predominant methodologies for embryo quality assessment are commonly used: morphological assessment and preimplantation genetic testing for aneuploidy (PGT‐A).

Morphological scrutiny remains a cornerstone methodology in the evaluation of embryo viability, bolstered by an extensive body of scientific literature. Existing literature suggests the relationship between the morphological assessment of a blastocyst and subsequent rates of implantation, pregnancy, and live births.^1^, ^2^, ^3^, ^4^, ^5^ In a similar vein, studies corroborate that blastocysts attaining requisite developmental milestones by Day 5 are generally associated with more favorable clinical outcomes as opposed to those reaching similar markers by Day 6.^6^, ^7^, ^8^ However, the accuracy of morphological evaluations is subject to certain limitations. Factors such as the skill level of the assessor and variations between laboratories can compromise the reliability of this assessment technique. Furthermore, intrinsic limitations to the methodology itself can pose challenges.^9^, ^10^, ^11^ Notably, existing data indicate that a considerable proportion—nearly half—of blastocysts, despite being graded high on morphological criteria, may be aneuploid.^12^, ^13^ Given that chromosomal aneuploidy is a significant contributor to the failure of IVF‐ET procedures, the data cast a shadow over the prudence of exclusive reliance on morphological criteria for assessment.^14^, ^15^

PGT‐A is a test that involves biopsying the trophectoderm (TE) cells of a blastocyst to analyze chromosomal count, thereby indicating whether the blastocyst is euploid or aneuploid. Several reports have indicated that PGT‐A can reduce the rate of miscarriage and improve live birth outcomes in specific cases such as recurrent implantation failure (RIF), recurrent pregnancy loss (RPL), and among patients of advanced maternal age.^16^, ^17^, ^18^, ^19^ Nevertheless, PGT‐A comes with its own set of constraints, including the intrinsic risks linked to invasive biopsy techniques, as well as the inability to guarantee successful live births even when transferring euploid blastocysts.

Each assessment approach has inherent weaknesses. Moreover, the extent to which these methods interact is not well‐understood. Therefore, additional rigorous investigation is warranted. Although there is a growing volume of research into the impact of morphological factors on the viability of euploid embryos, the findings to date are not definitive. Some studies, such as those by Capalbo and others, suggest that pregnancy outcomes do not differ significantly between morphologically low‐grade and high‐grade embryos if they are euploid.^20^, ^21^ On the other hand, several other studies assert that even euploid embryos with low‐grade morphology yield worse pregnancy outcomes compared to those with high‐grade morphology.^6^, ^7^, ^22^, ^23^, ^24^, ^25^ The inconsistency in current research underscores the need for more definitive studies on the issue. Consequently, our study aims to address these gaps in the current body of literature by conducting a comprehensive analysis of both morphological and chromosomal factors affecting pregnancy outcomes. Our primary objective is to evaluate how morphological quality impacts pregnancy outcomes of euploid embryos determined by PGT‐A. As a secondary objective, we will also identify which specific aspects of morphological assessment exert the most significant impact on pregnancy outcomes, offering valuable insights that could inform both clinical practice and patient decisions.

## 2 MATERIALS AND METHODS

### 2.1 Inclusion criteria

We conducted a retrospective analysis of patients at our clinic who underwent PGT‐A and had blastocyst transfer from April 2020 to September 2022.

During the study period, patients underwent treatment through IVF or intracytoplasmic sperm injection (ICSI), and if the embryos were successfully cultured up to the blastocyst stage, they were then cryopreserved using vitrification.

The criteria for PGT‐A were limited to couples with normal karyotypes who had either experienced two consecutive failures in IVF‐ET (referred to as RIF) or had a history of RPL.

Throughout the course of assisted reproductive technology (ART) treatments, patients underwent routine ultrasonographic assessments. Individuals with severe uterine pathologies capable of altering the uterine cavity morphology, such as substantial myomas or adenomyosis, were excluded from the study. Patients diagnosed with submucosal myomas underwent myomectomy prior to embryo transfer.

### 2.2 Ovarian stimulation protocol

Based on factors such as age, anti‐Müllerian hormone (AMH), antral follicle count (AFC), and basal follicle‐stimulating hormone (FSH) levels, ovarian stimulation was conducted using either the mild stimulation protocol or the antagonist protocol, in accordance with the woman's ovarian reserve.

In the mild stimulation protocol, treatment began with clomiphene citrate (Clomid, 50 mg; Sanofi‐Aventis) from days 2–4 of menstruation. Regular monitoring of the diameter of the follicle, E2, FSH, and LH levels was carried out. If necessary, human menopausal gonadotropin (HMG; Ferring Pharmaceuticals or ASKA Pharmaceutical) was administered at a dose of 150 IU. For oocyte maturation, final follicle growth and maturation were induced by administering hCG at a dose of 10 000 IU.

In the antagonist protocol, treatment was initiated between the 2–4 days of menstruation using either human menopausal gonadotropin (HMG; Ferring Pharmaceuticals) or recombinant FSH (Gonal‐f®; Merck Serono). The HMG dosage was adjusted based on the follicle diameter and E2, FSH, and LH levels. When the follicle diameter reached 10–14 mm, a GnRH antagonist, either ganirelix (Ganirest®, MSD) or cetrorelix (Cetrotide®, Merck Serono), was administered at a dose of 0.25 mg. Final follicular maturation was induced using a GnRH agonist (Lupron, Abbott Pharmaceuticals) alone, or combined with hCG (2000–5000 IU) in a dual trigger, depending on factors such as E2 levels, the number of follicles, and patient age. Patients underwent a transvaginal ultrasound‐guided oocyte retrieval procedure under intravenous sedation 35–37 h after receiving the trigger.

### 2.3 Laboratory protocol

Embryos were cultured in a time‐lapse imaging incubator (CCM‐iBIS NEXT; Astec) that acquired images at 15‐min intervals. Blastocysts were evaluated by embryologists according to the Gardner scoring criteria,^3^ focusing on the degree of expansion, Inner Cell Mass (ICM), and TE morphology. Each blastocyst is evaluated by multiple embryologists, with a primary evaluator assigned to each embryo. To ensure consistent and accurate assessments, our clinic's embryologists adhere to a strict protocol and undergo regular training sessions. These sessions emphasize practical evaluations using images of the same embryos by both experienced and trainee embryologists. This approach is reported to enhance consistency through image‐based assessments and consensus building.^26^, ^27^

In this study, we define those that received a “C” evaluation for either ICM or TE as belonging to the morphologically low‐grade (ML) group, and the remaining blastocysts as belonging to the morphologically high‐/average‐grade (MHA) group.

The obtained blastocysts were individually vitrified and cryopreserved using Kitazato vitrification media (Japan). After freezing the blastocysts, patients were counseled to determine the number of blastocysts to be tested with PGT‐A.

Biopsy of blastocyst‐stage embryos was performed in a drop of about 20–30 μL of human tubal fluid (HTF) medium supplemented with HEPES (SW012, Nakamedical, Inc, Tokyo, Japan). Blastocysts were held in a holding pipette and irradiated with a 0.22 ms laser pulse (Saturn 5™ Active; Cooper Surgical, Trumbull, USA) to open the zona pellucida. Four to six TE cells were aspirated from the newly opened zona pellucida with a biopsy pipette (15118, Vitrolife, Gothenburg, Sweden), and the laser pulse was applied three times where the TE cells were stretched. The TE cells were then severed using a mechanical “flicking” method. The harvested TE cells were washed in phosphate‐buffered saline (PBS) containing 1% polyvinyl pyrrolidone and transferred to 0.2‐mL PCR tubes containing 1.5 μL of PBS. Samples were stored at −30°C until analysis. New instruments were used for each sample to prevent DNA contamination between samples.

As of October 2021, OVUS, the company to which we outsource PGT‐A analysis, has changed the instruments and kits used for PGT‐A. Therefore, the analysis methods used are shown below.

Analysis methods used by OVUS until September 30, 2021: Frozen samples were transported to the analysis company (OVUS Co., Ltd, Aichi, Japan) for whole‐genome amplification from TE cells using the SurePlex WGA Kit (Illumina, San Diego, CA). Nextera libraries were prepared from the amplified DNA according to the manufacturer's protocol, and sequencing was performed using the VeriSeq PGS assay system with MiSeq (Illumina). The sequencing data were analyzed with BlueFuse Multi analysis software v4.5 to determine chromosomal copy number changes.

Analysis methods used by OVUS from October 1, 2021, onwards: Frozen samples were transported to the analysis company (OVUS Co., Ltd) for whole‐genome amplification from TE cells using the Ion ReproSeq PGS Kit (Thermo Fisher Scientific). DNA libraries were prepared from the amplified whole genome using the Chef System (Thermo Fisher Scientific) and sequenced using Ion GeneStudio S5 (Thermo Fisher Scientific) according to the manufacturer's protocols. The obtained sequencing data were analyzed using Ion Reporter software (Thermo Fisher Scientific) to determine chromosomal copy number changes.

Blastocysts diagnosed as euploid were then transferred. If multiple euploid embryos were present, transfers were done in order of best morphological grade first.

For all transfer cycles, a hormone replacement therapy cycle was used. Estrogen supplementation was started on menstrual cycle days 2–3 using two transdermal estradiol patches (Estrana®, Hisamitsu Pharmaceutical) of 0.72 mg each, every other day.

On menstrual cycle days 9–11, the endometrial thickness was measured by transvaginal ultrasound. If the endometrium was 7 mm or more, the estradiol patches were increased to a maximum of eight patches, two every other day, and the day after the increase to eight patches was considered the estimated ovulation day.

If the endometrium was less than 7 mm, the administration of estradiol patches was extended by about 1 week, and the endometrial thickness was measured again.

Luteal phase support was initiated from the expected ovulation day using chlormadinone acetate (Lutoral®, Fuji Pharma) 2 mg three times a day, and the day of frozen–thawed embryo transfer was determined. Subsequently, the estradiol patches were reduced to 4 and continued every other day.

Pregnancy was evaluated by measuring serum β‐hCG on Day 17 after the expected ovulation day. β‐hCG ≧1.0 mIU/mL was considered implantation‐positive, and transvaginal ultrasound was performed 1 week later to confirm the presence of an intrauterine gestational sac. Clinical pregnancy was defined as the presence of an intrauterine gestational sac on transvaginal ultrasound. Luteal phase support was continued until 9 weeks and 5 days of pregnancy.

### 2.4 Clinical outcome

The primary objective of this study is to assess the impact of morphological quality on pregnancy outcomes—including implantation rate (IR), pregnancy rate (PR), live birth rate (LBR), and miscarriage rate (MR)—in embryos determined to be euploid by PGT‐A. Specifically, we aim to compare these pregnancy outcomes between embryos classified as MHA and ML based on established criteria.

The secondary objective focuses on identifying which specific aspects of morphological evaluation—such as the degree of blastocyst expansion, quality of the ICM, and TE morphology—have the most significant impact on pregnancy outcomes. The objective is to discern which among these factors is most strongly correlated with successful live births.

### 2.5 Statistical analysis

Statistical calculations were performed using statistical analysis software R (version 4.3.0). Continuous variables (such as age, number of miscarriages, and total number of transfers) in the MHA and ML were checked for non‐normal distribution using the Shapiro–Wilk test and analyzed using the Mann–Whitney U test. Comparisons of implantation rate, pregnancy rate, live birth rate, and miscarriage rate between the MHA and ML groups were analyzed with Pearson's *χ*2 test. Logistic regression analysis was performed on ICM, TE, blastocoele expansion, and blastocyst development speed to investigate which morphological evaluations most affected LBR. The significance level for statistical analysis was set at 0.05.

## 3 RESULTS

During the study period, 4535 blastocysts underwent PGT‐A testing. Of these, 1124 (24.8%) were classified as euploid, 550 (12.1%) as mosaic, and 2810 (62.0%) as aneuploid. Amplification failed in 51 biopsied blastocysts (1.12%), yielding no genetic results.

A total of 591 cycles included potentially transferable blastocysts (either euploid or mosaic embryos). The following cycles were excluded:

- 86 cycles with mosaic embryo transfers.
- 32 cycles in which embryos were transferred from other facilities and pre‐freezing morphological evaluation could not be performed at our clinic.
- 21 cycles with the presence of severe adenomyosis or myomas that could distort the uterine cavity.
- 1 cycle where the patient voluntarily withdrew from the study.

After these exclusions, 451 cycles remained for the final analysis, involving 303 patients. The distribution of these patients was as follows:

- 197 had a single embryo transfer.
- 74 had two transfers.
- 26 had three transfers.
- 5 had four transfers.
- 1 had eight transfers.

**FIGURE 1:**
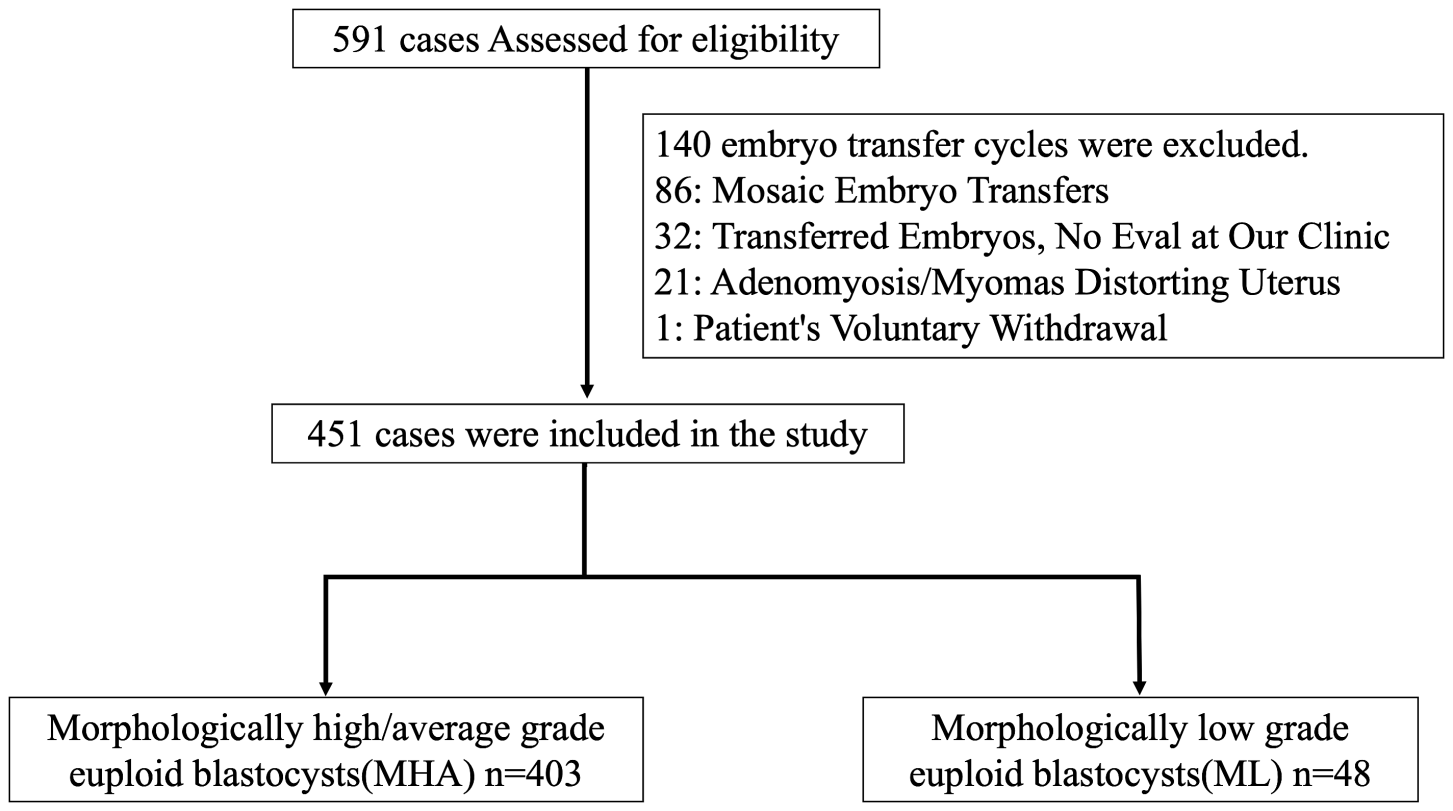
Enrollment, assignment, and analysis of cycles in which PGT‐A‐tested embryos were transferred.

Of the 451 cycles analyzed, 403 were in the MHA group and 48 were in the ML group (Figure 1).

Characteristics of the treatment cycles for the MHA and ML groups are presented in Table 1.

**TABLE 1:** Baseline characteristics of the MHA and ML groups.^a^

Characteristic	MHA (*n* = 403)	ML (*n* = 48)	*p* value
Age at retrieval (Median [IQR])^b^	37 (34–40)	36 (34–39)	0.378
Age at transfer (Median [IQR])	38 (35–40)	38 (34–40)	0.284
Gravidity (Median [IQR])	2 (1–3)	2 (0–2)	0.310
Parity (Median [IQR])	0 (0–1.0)	0 (0–0)	0.759
Prior miscarriage (Median [IQR])	1 (0–2)	2 (1–3)	0.312
Number of total ET cycles (Median [IQR])	7 (5–9.5)	7 (5–10)	0.573
Indication of PGT‐A (RIF/RPL)^c^	246/157	25/23	0.297

^a^ Morphologically high‐/average‐ and morphologically low‐grade groups.

^b^ The data are represented as median (interquartile range [IQR]).

^c^ Indication of preimplantation genetic testing for aneuploidies (PGT‐A). RIF represents recurrent implantation failure, and RPL represents recurrent pregnancy loss. The data are represented as the number of cases for RIF/RPL.

No significant differences were observed between the two groups in terms of age at retrieval, age at transfer, pregnancy history, birth history, number of miscarriages, total number of transfers including the current cycle, or PGT‐A indications.

**FIGURE 2:**
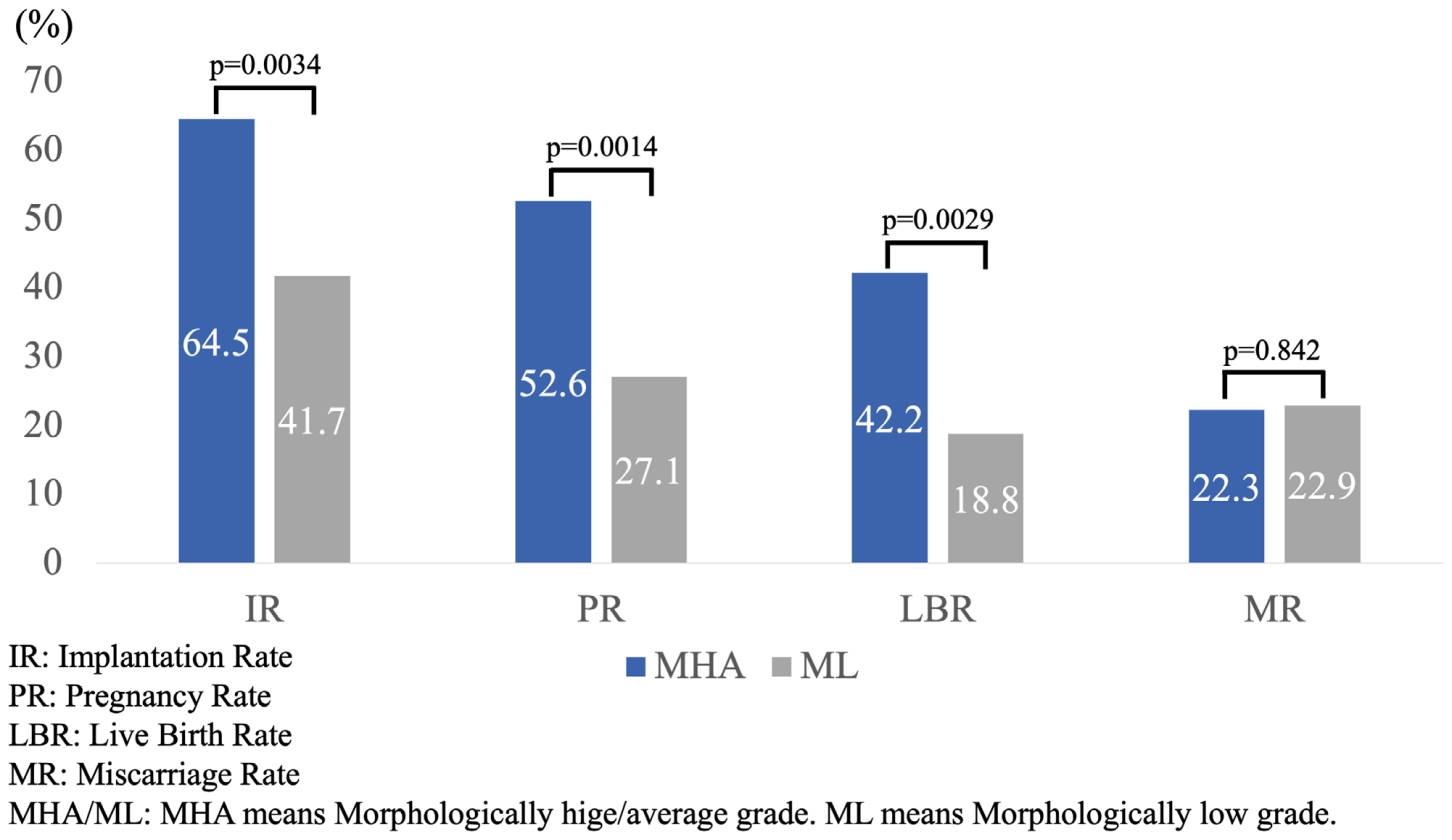
Comparison of pregnancy outcomes between the MHA and ML groups.

Pregnancy outcomes for the MHA and ML groups are displayed in Figure 2. In the MHA group, IR was 64.5%, PR was 52.6%, and LBR was 42.2%. For the ML group, these rates were 41.7%, 27.1%, and 18.8%, respectively. In all aspects—implantation, clinical pregnancy, and live birth—the MHA group significantly outperformed the ML group. In terms of MR, both the MHA and ML groups showed similar results, with rates of 22.3% and 22.9%, respectively.

To investigate which factors among the morphological evaluations (ICM, TE, blastocoele expansion, and blastocyst development speed) affected the LBR, a multivariate logistic regression analysis was performed, adjusting for confounding factors such as age at retrieval, pregnancy history, birth history, total number of transfers, and PGT‐A indications. The results are presented in Table 2. When the ICM was rated as “A,” the LBR was 46%. Using this as a benchmark, a “B” rating for ICM resulted in an LBR of 22%, with an odds ratio of 0.40 (*p* = 0.001), significantly lower than the benchmark. The LBR for a “C” ICM rating was 10% with an odds ratio of 0.17 (*p* = 0.107), which did not reach statistical significance.

**TABLE 2:** Multiple logistic regression analysis of the association between the live birth rate and the morphological evaluation of blastocysts.

Variable	Value	LBR^a^ (%)	Odds ratio (OR)	95% confidence interval (CI)	*p* value*
Inner cell mass (ICM) grade	A	157/344 (46)	–	–	–
	B	21/97 (22)	0.40	0.22–0.71	0.001
	C	1/10 (10)	0.17	0.02–1.46	0.107
Trophectoderm (TE) grade	A	52/117 (44)	–	–	–
	B	119/296 (40)	1.12	0.70–1.79	0.649
	C	8/38 (21)	0.69	0.27–1.77	0.439
Day of blastocyst vitrification	5	151/355 (43)	–	–	–
	6	28/96 (29)	0.65	0.37–1.11	0.116
Blastocyst expansion grade	5	2/3 (67)	–	–	–
	4	131/304 (43)	0.24	0.02–2.97	0.266
	3	46/144 (32)	0.18	0.01–2.18	0.174

*Note*: The odds ratio, 95% confidence interval, and *p* value are provided for each comparison to the reference category (denoted by “–”).

Outcome adjusted for the following confounding variables: maternal age at retrieval, gravidity, parity, number of total ET cycles, and indication of PGT‐A.

^a^ Live birth rate: The number of successful live births per total cases, expressed as a percentage.

* Statistically significant at the 0.05 level.

The LBR for an “A” TE rating was 44%, with a “B” rating leading to an LBR of 40% and an odds ratio of 1.12 (*p* = 0.649), while a “C” rating resulted in an LBR of 21% and an odds ratio of 0.69 (*p* = 0.439). None of these achieved statistical significance.

The LBR for blastocysts on Day 5 was 43%. Using this as a reference, the rate on Day 6 was 29%, with an odds ratio of 0.65 (*p* = 0.116), which did not achieve statistical significance. When the blastocyst expansion grade was 5, the LBR was 67%. Using this as a reference, for grade 4, the LBR was 43%, with an odds ratio of 0.24 (*p* = 0.266), and for grade 3, the rate was 32%, with an odds ratio of 0.18 (*p* = 0.174). None of these achieved statistical significance.

These results suggest that among euploid embryos, the morphological evaluation of the ICM has the most significant impact on the LBR.

## 4 DISCUSSION

This study investigated the impact of morphological assessment on pregnancy outcomes across 451 cycles of single euploid embryo transfers. The findings revealed that the pregnancy outcomes were significantly lower when using morphologically low‐grade euploid embryos compared to those with high‐grade morphological qualities. Additionally, no significant difference was observed in the miscarriage rate between embryos with high‐ and low‐grade morphology. Furthermore, it was confirmed that the morphological evaluation of the ICM had a high correlation with LBR.

While there are reports suggesting that embryos with good morphology are more likely to be euploid,^13^, ^20^, ^21^, ^28^ it is also known that about half of such good‐quality embryos are diagnosed as aneuploid.^12^, ^13^ Furthermore, it is a fact that even when transferring embryos diagnosed as euploid via PGT‐A, it does not always result in a live birth. This suggests that the evaluation of an embryo as either euploid or aneuploid is only capturing one aspect of the embryo's quality. Morphological assessment, on the other hand, reflects attributes of the embryo that cannot be fully gauged by chromosome number alone.

In embryos where PGT‐A is not performed, pre‐freeze TE evaluation is reported to have the highest correlation with LBR.^29^, ^30^ The subjectivity in TE evaluations, which are morphological in nature, could potentially be minimized by incorporating PGT‐A, as it involves a biopsy of TE cells for objective chromosomal evaluation. In euploid embryos as determined by PGT‐A, the ICM, which is not evaluated through biopsy in PGT‐A, is believed to have a significant impact on LBR.

This study highlights the utility of combining morphological assessments with chromosomal analysis for embryo selection. Specifically, by integrating information obtained from PGT‐A with morphological evaluations, particularly of the ICM, more accurate embryo selection can be achieved. This combination may lead to higher live birth rates and increase the likelihood of successful treatment outcomes.

Several studies have already reported that transferring morphologically high‐grade euploid embryos results in better pregnancy outcomes, a finding that our study supports.^6^, ^7^, ^22^, ^23^, ^24^, ^25^ However, there are also some reports stating that pregnancy outcomes do not differ whether the transferred euploid embryo is assessed as high or low grade morphologically.^20^, ^21^

Several reasons are considered for these conflicting research results:

1. Morphological assessments inherently involve subjectivity. This means that variations in evaluator skill and facility‐specific assessment methods can result in inconsistent morphological evaluations.
2. When multiple euploid embryos are available, clinicians are more likely to choose those with high‐grade morphology for transfer. As a result, the sample size of transferred euploid embryos with low‐grade morphology tends to be small in most studies. In our research, there were 403 cases of high‐grade morphology versus 48 cases of low‐grade morphology, a significant discrepancy in sample sizes.
3. In this study, we excluded cases with severe uterine myomas or adenomyosis; however, there are other factors such as chronic endometritis and unidentified elements that may inhibit implantation. Ideally, future research should adjust for these external factors to investigate the impact of morphological assessment on euploid embryos, but currently, this is challenging to accomplish.

Our study has several limitations. First, the number of cycles where low‐grade morphology embryos were transferred (48 cycles) is relatively small. A larger sample size may reveal significant differences in evaluation items other than the ICM, such as the TE, blastocoele expansion, and blastocyst development speed. Second, only Japanese patients were included in the study, which may limit the generalization of our findings to other ethnic groups. Thus, further research is needed to generalize our findings. Our facility plans to gather more data and conduct additional studies, aiming to enhance the generalizability and robustness of our conclusions.

## 5 CONCLUSION

The pregnancy outcome of blastocysts with low‐grade morphological evaluation was estimated to be significantly lower than that of blastocysts with high‐grade morphological evaluation in euploid embryos. The results also suggest that ICM evaluation has the greatest influence on the live birth rate among the morphological evaluations of euploid embryos.

## CONFLICT OF INTEREST STATEMENT

The authors declare that there are no conflicts of interest.

## HUMAN RIGHTS STATEMENTS

All procedures were carried out in compliance with the ethical standards of the relevant committees on human experimentation, both institutional and national, as well as the 1964 Declaration of Helsinki and its subsequent amendments.

## INFORMED CONSENT

This research is a retrospective study targeting patients who had submitted informed consent for undergoing fertility treatment at our clinic. The opt‐out approach was employed to recruit research participants; this involves providing potential participants with information about the research on our clinic's website and including them as research subjects unless they expressly state their refusal to participate.

## ETHICS STATEMENT

The research design was approved by the Ethics Committee of the Institutional Review Board of Asada Ladies Clinic (approval number: 2022–17).

## ANIMAL STUDIES

This article does not contain any study with animal participants that have been performed by the author.

## References

[1] Bakkensen JB , Brady P , Carusi D , Romanski P , Thomas AM , Racowsky C . Association between blastocyst morphology and pregnancy and perinatal outcomes following fresh and cryopreserved embryo transfer. J Assist Reprod Genet. 2019;36(11):2315–2324. 31512049 10.1007/s10815-019-01580-0 PMC6885471

[2] Balaban B , Urman B , Sertac A , Alatas C , Aksoy S , Mercan R . Blastocyst quality affects the success of blastocyst‐stage embryo transfer. Fertil Steril. 2000;74:282–287. 10927045 10.1016/s0015-0282(00)00645-2

[3] Gardner DK , Lane M , Stevens J , Schlenker T , Schoolcraft WB . Blastocyst score affects implantation and pregnancy outcome: towards a single blastocyst transfer. Fertil Steril. 2000;73:1155–1158. 10856474 10.1016/s0015-0282(00)00518-5

[4] Racowsky C , Combelles CM , Nureddin A , Pan Y , Finn A , Miles L , et al. Day 3 and day 5 morphological predictors of embryo viability. Reprod Biomed Online. 2003;6:323–331. 12735868 10.1016/s1472-6483(10)61852-4

[5] Hill MJ , Richter KS , Heitmann RJ , Graham JR , Tucker MJ , DeCherney AH , et al. Trophectoderm grade predicts outcomes of single‐blastocyst transfers. Fertil Steril. 2013;99(5):1283–1289. 23312233 10.1016/j.fertnstert.2012.12.003

[6] Irani M , O'Neill C , Palermo GD , Xu K , Zhang C , Qin X , et al. Blastocyst development rate influences implantation and live birth rates of similarly graded euploid blastocysts. Fertil Steril. 2018;110(1):95–102. 29908774 10.1016/j.fertnstert.2018.03.032

[7] Lou H , Li N , Guan Y , Zhang Y , Hao D , Cui S . Association between morphologic grading and implantation rate of euploid blastocyst. J Ovarian Res. 2021;14(1):18. 33485390 10.1186/s13048-021-00770-8 PMC7827997

[8] Wang X , Zhen J , Sun Z , Yu Q , Deng C , Zhou Y , et al. Effects of fifth day (D5) or sixth day (D6) frozen‐thawed blastocysts on neonatal outcomes. Zygote. 2016;24(5):684–691. 27587093 10.1017/S0967199415000696

[9] Bendus AEB , Mayer JF , Shipley SK , Catherino WH . Interobserver and intraobserver variation in day 3 embryo grading. Fertil Steril. 2006;86(6):1608–1615. 17074349 10.1016/j.fertnstert.2006.05.037

[10] Sundvall L , Ingerslev HJ , Knudsen UB , Kirkegaard K . Inter‐ and intra‐observer variability of time‐lapse annotations. Hum Reprod. 2013;28(12):3215–3221. 24070998 10.1093/humrep/det366

[11] Ruiz de Assín R , Clavero A , Gonzalvo MC , Ramírez JP , Zamora S , Fernández A , et al. Comparison of methods to determine the assigned value in an external quality control programme for embryo evaluation. Reprod Biomed Online. 2009;19(6):824–829. 20031024 10.1016/j.rbmo.2009.09.026

[12] Alfarawati S , Fragouli E , Colls P , Stevens J , Gutierrez‐Mateo C , Schoolcraft WB , et al. The relationship between blastocyst morphology, chromosomal abnormality, and embryo gender. Fertil Steril. 2011;95:520–524. 20537630 10.1016/j.fertnstert.2010.04.003

[13] Li N , Guan Y , Ren B , Zhang Y , Du Y , Kong H , et al. Effect of blastocyst morphology and developmental rate on euploidy and live birth rates in preimplantation genetic testing for aneuploidy cycles with single‐embryo transfer. Front Endocrinol (Lausanne). 2022;13:858042. 35498424 10.3389/fendo.2022.858042 PMC9044033

[14] Werner M , Reh A , Grifo J , Perle MA . Characteristics of chromosomal abnormalities diagnosed after spontaneous abortions in an infertile population. J Assist Reprod Genet. 2012;29:817–820. 22618194 10.1007/s10815-012-9781-3 PMC3430785

[15] Scott RT Jr , Ferry K , Su J , Tao X , Scott K , Treff NR . Comprehensive chromosome screening is highly predictive of the reproductive potential of human embryos: a prospective, blinded, nonselection study. Fertil Steril. 2012;97:870–875. 22305103 10.1016/j.fertnstert.2012.01.104

[16] Bhatt SJ , Marchetto NM , Roy J , Morelli SS , McGovern PG . Pregnancy outcomes following in vitro fertilization frozen embryo transfer (IVF‐FET) with or without preimplantation genetic testing for aneuploidy (PGT‐A) in women with recurrent pregnancy loss (RPL): a SART‐CORS study. Hum Reprod. 2021;36(8):2339–2344. 34027546 10.1093/humrep/deab117

[17] Zheng Z , Tan J , Chen L , Liu S , Zhou C , Li Y . PGT‐A improved singleton live birth rate among all age groups of women who underwent elective single blastocyst transfer: a single‐centre retrospective study. J Assist Reprod Genet. 2023;40(6):1417–1427. 37055598 10.1007/s10815-023-02775-2 PMC10310591

[18] Pantou A , Mitrakos A , Kokkali G , Petroutsou K , Tounta G , Lazaros L , et al. The impact of preimplantation genetic testing for aneuploidies (PGT‐A) on clinical outcomes in high risk patients. J Assist Reprod Genet. 2022;39(6):1341–1349. 35338417 10.1007/s10815-022-02461-9 PMC9174385

[19] Iwasa T , Kuwahara A , Takeshita T , Taniguchi Y , Mikami M , Irahara M . Preimplantation genetic testing for aneuploidy and chromosomal structural rearrangement: a summary of a nationwide study by the Japan Society of Obstetrics and Gynecology. Reprod Med Biol. 2023;22(1):e12518. 37274391 10.1002/rmb2.12518 PMC10233076

[20] Capalbo A , Rienzi L , Cimadomo D , Maggiulli R , Elliott T , Wright G , et al. Correlation between standard blastocyst morphology, euploidy and implantation: an observational study in two centers involving 956 screened blastocysts. Hum Reprod. 2014;29:1173–1181. 24578475 10.1093/humrep/deu033

[21] Viñals Gonzalez X , Odia R , Naja R , Serhal P , Saab W , Seshadri S , et al. Euploid blastocysts implant irrespective of their morphology after NGS‐(PGT‐A) testing in advanced maternal age patients. J Assist Reprod Genet. 2019;36(8):1623–1629. 31165389 10.1007/s10815-019-01496-9 PMC6707991

[22] Irani M , Reichman D , Robles A , Melnick A , Davis O , Zaninovic N , et al. Morphologic grading of euploid blastocysts influences implantation and ongoing pregnancy rates. Fertil Steril. 2017;107(3):664–670. 28069172 10.1016/j.fertnstert.2016.11.012

[23] Zhang WY , Johal JK , Gardner RM , Bavan B , Milki AA . The impact of euploid blastocyst morphology and maternal age on pregnancy and neonatal outcomes in natural cycle frozen embryo transfers. J Assist Reprod Genet. 2022;39(3):647–654. 35122177 10.1007/s10815-022-02423-1 PMC8995226

[24] Shear MA , Vaughan DA , Modest AM , Seidler EA , Leung AQ , Hacker MR , et al. Blasts from the past: is morphology useful in PGT‐A tested and untested frozen embryo transfers? Reprod Biomed Online. 2020;41(6):981–989. 33011085 10.1016/j.rbmo.2020.07.014 PMC7872471

[25] Peng X , Yu M , Li L , Fu W , Chen H , Sun X , et al. Effects of euploid blastocyst morphological development on reproductive outcomes. Reprod Biol. 2020;20(4):496–500. 32861682 10.1016/j.repbio.2020.08.002

[26] Castilla JA , Ruiz de Assín R , Gonzalvo MC , Clavero A , Ramírez JP , Vergara F , et al. External quality control for embryology laboratories. Reprod Biomed Online. 2010;20(1):68–74. 20158990 10.1016/j.rbmo.2009.09.033

[27] Ruiz de Assin R , Clavero A , Gonzalvo MC , Rosales A , Zamora S , Martinez L , et al. Reducing inter‐observer variability in embryo evaluation by means of training courses. J Assist Reprod Genet. 2011;28:1129–1133. 21947757 10.1007/s10815-011-9639-0 PMC3224171

[28] Majumdar G , Majumdar A , Verma IC , Upadhyaya KC . Relationship between morphology, euploidy and implantation potential of cleavage and blastocyst stage embryos. J Hum Reprod Sci. 2017;10(1):49–57. 28479756 10.4103/0974-1208.204013 PMC5405648

[29] Ebner T , Tritscher K , Mayer RB , Oppelt P , Duba HC , Maurer M , et al. Quantitative and qualitative trophectoderm grading allows for prediction of live birth and gender. J Assist Reprod Genet. 2016;33:49–57. 26572782 10.1007/s10815-015-0609-9 PMC4717145

[30] Honnma H , Baba T , Sasaki M , Hashiba Y , Ohno H , Fukunaga T , et al. Trophectoderm morphology significantly affects the rates of ongoing pregnancy and miscarriage in frozen‐thawed single‐blastocyst transfer cycle in vitro fertilization. Fertil Steril. 2012;98:361–367. 22682029 10.1016/j.fertnstert.2012.05.014

